# Risk factors for falls in older adults with diabetes mellitus: systematic review and meta-analysis

**DOI:** 10.1186/s12877-024-04668-0

**Published:** 2024-02-28

**Authors:** Larissa Barros Freire, Joaquim Pereira Brasil-Neto, Marianne Lucena da Silva, Milena Gonçalves Cruz Miranda, Lorrane de Mattos Cruz, Wagner Rodrigues Martins, Leonardo Petrus da Silva Paz

**Affiliations:** 1 Postgraduate course in Health Sciences and Technologies, University of Brasília (UnB) - Campus Ceilândia, Brasília, DF Brazil; 2 Faculty of Medicine, Centro Universitário Euro-Americano (Unieuro), Brasília, Brazil; 3 Department of Collective Health, University of Brasilia, Brasília, Brazil; 4 Graduate program of Physical Therapy, University of Brasilia - Campus Ceilândia, Brasília, Brazil; 5 University of Brasilia, Faculty of Ceilândia, Rehabilitation Sciences and Physical Education Postgraduate Program, Brasília, DF Brazil; 6 University of Brasilia, Campus Ceilandia – Faculty of Ceilandia, Brasília, 72220-275 Brazil

**Keywords:** Type 2 diabetes mellitus, Falls, Risk of falls, Older adults, Accidental falls

## Abstract

**Aim:**

To identify risk factors for falls in older adults with Type 2 Diabetes Mellitus (T2DM).

**Methods:**

The eligible studies identified factors associated with the risk of falls in older adults with T2DM. We searched PubMed, Cinahl, Web of Science, Scopus, and the Cochrane Library databases. The review has been updated and the last review date was November 30, 2023 (CRD42020193461).

**Results:**

Twelve studies met the inclusion criteria, and eight studies were included in the meta-analysis. These studies included a total of 40,778 older adults with T2DM, aged 60 to 101 years. The risk of developing the outcome falls in older adults with T2DM is 63% higher compared to the risk in older adults without T2DM (HR 1.63; 95% CI [1.30 - 2.05]). The overall chance of falling in older adults with T2DM is 59% higher than that of non-diabetic older adults (OR 1.59; 95% CI [1.36 -1.87]), and in older adults with T2DM who take insulin the chance of falling is 162% higher (OR 2.62; 95% CI [1.87 - 3.65]). No results on diabetic polyneuropathy were found in the studies.

**Conclusion:**

Older adults with T2DM present a higher risk of falls compared to non-diabetics. Among the included older adults with T2DM, the most important factor associated with a higher risk of falls was insulin use.

**Trial registration:**

Registered in the International Prospective Register of Systematic Reviews (CRD42020193461).

**Supplementary Information:**

The online version contains supplementary material available at 10.1186/s12877-024-04668-0.

## Background

Diabetes Mellitus (DM) is a chronic disease that presents as a complex metabolic disorder, whose main characteristic is hyperglycemia [1, 2]. This characteristic results from a deficiency in the secretion of insulin, which is a hormone produced by pancreatic β-cells, which has the primary function of maintaining glucose homeostasis [3]. More than 90% of people with DM in the world are type 2. The complications of the disease can reduce life expectancy significantly [4], with DM being the ninth leading cause of death worldwide, and approximately 1 in 11 adults are diagnosed with the disease. The main causes of DM are increased obesity, high energy-density diets, an aging population, unhealthy eating habits, and sedentary lifestyles [5].

The main complications of DM are retinopathy and polyneuropathy. Diabetic retinopathy causes vision loss and is associated with poor glycemic control and prolonged duration of the disease [6]. Diabetic polyneuropathy (DPN) is associated with poor quality of life due to the frequent occurrence of neuropathic pain and ulcers of the feet [7].

DPN is suggested to be a potential risk factor for falls [8]. Damage to the peripheral nervous system is classified as diabetic neuropathy. The main symptoms of DPN are numbness, insensitivity to injury, loss of postural stability, and intractable neuropathic pain [9].

Due to these symptoms of DPN, falls in older adults represent a public health problem, particularly as it is thought that the incidence of falls can be as high as 40% in diabetic older adults. The increased risk of falls among older adults with T2DM may be associated with the presence of neuropathy and retinopathy [10]. Proper management of risk factors for falls in this population is of the utmost importance, as falls are a leading cause of fatal and non-fatal injuries among people aged 65 and over [11, 12], with 30% of people over 60 presenting a fall in any given year [13]. Falls are commonly defined as "an event during which a person unintentionally moves their body to a lower level or the ground " [14].

The scientific literature in the area suffers from a paucity of studies investigating factors associated with the risk of falls in older adults with DM. The lack of studies is even more evident in the older adult diabetic population with DPN [8, 15–18]. In addition, studies on the risk factors for falls in older adults with DM have reported heterogeneous results [19–21]. One of the causes for this heterogeneity may be the fact that the risk of falls has generally been investigated using data derived from samples containing younger and older adults in the same cohort. In addition, from a data analysis point of view, the authors presented the results mixing Odds and Hazard Ratios [10].

The objective of the present systematic review was to compare the incidence of falls in older adults with and without T2DM, as well as to verify whether older adults with diabetes on insulin or with polyneuropathy have a higher risk of falls than older adults without diabetes.

## Methods

The present systematic review was conducted according to the criteria of the Preferred Reporting Items for Systematic Reviews and Meta-Analyses (PRISMA) [22] and registered in the International Prospective Register of Systematic Reviews (CRD42020193461).

The construction of the research question was based on the following PICO strategy. Population: men and women aged 60 years or older diagnosed with type 2 diabetes mellitus; Intervention: not applicable; Control: diabetics without diabetic polyneuropathy or non-diabetic older adults; Outcome: number of falls through questionnaires and/or self-reports; or the risk or chance of falls. The risk of falls could also be assessed by means of cut-off points of mobility, body balance, and gait instruments through functional tests.

### Data sources and search strategy

Relevant articles published from inception to July 2021 were searched in five databases (PubMed, Cinahl, Web of Science, Scopus, and Cochrane Library). The search was updated in March 2023 (See Additional file 1: Table S1). There was no restriction imposed on the year of publication. A manual search for the articles was also performed.

The following keywords and search strategy were used for the electronic search and adapted for each database as necessary. We used a combination of the following terms: aged, diabetes mellitus type 2, accidental falls, aged AND diabetes mellitus type 2, aged AND diabetes mellitus type 2, AND accidental falls.

### Study selection and inclusion criteria

Studies with samples containing exclusively older adults, aged over 60 years, and clinically diagnosed with type 2 diabetes mellitus, with or without polyneuropathy and retinopathy, were selected. Diabetic participants can experience a combination of symptoms/signs that may include one or more of the following: reduced sensation in the soles of the feet, hyper- or hypoglycemia, and risk of falls or actual falls.

Studies considering older adults under the age of 60 years were included if data from the older adults were presented separately in the text or table. We included observational studies, prospective and retrospective cohorts, and cross-sectional studies in this review.

The articles considered for this review met the following criteria: cohort and cross-sectional studies published in English, Spanish, or Portuguese; and studies that identify the risk of falls or falls in older adults with T2DM. Retrospective studies were considered when data were available regarding the classification of older adults as fallers and non-fallers.

Articles that presented one of the following characteristics were excluded: duplicate articles, data from unpublished studies, studies with designs that do not enable assessment of the risk or chance of falling, studies with incomplete methodology (no diagnosis of T2DM, lack of data on number of falls and T2DM), when the sample did not include older adults, as well as studies that did not present data on the ages and risk of falls of adults and older adults separately; and finally, we excluded studies which did not assess the risk of falls.

The studies were selected in two phases. In the first phase, they were evaluated by title and abstract, and in the second phase, the full text of the pre-selected studies was read to confirm eligibility. The selection process was carried out through the Rayyan platform (https://rayyan.qcri.org).

### Data extraction and quality assessment

The data summary was performed using a spreadsheet built in Microsoft® Office Excel®, by each evaluator separately. The summarized results were compiled and presented in tables. Columns included the title, author, date, objective, type of study, type of participants, patient demographics, type of experiment, outcome, and quality score. All studies were assessed using the Newcastle-Ottawa tool [23, 24]. This tool includes 8 items, divided into 3 domains: selection, comparison, and exposure or outcome. The risk of bias was assessed independently by two authors (LBF, MLS) using the Newcastle-Ottawa scale. The third reviewer (LPSP) resolved apparent discrepancies in the Quality Assessment and Risk of Bias process.

### Data overview and analysis

The meta-analysis was performed using 8 of the 12 studies selected in the systematic review. The reasons for the exclusion of these 4 studies were as follows: 3 articles had OR and HR with divergent data and in one article the study data were not provided by the authors for meta-analysis. Analyses with OR and HR data were performed separately for each outcome. The logarithm calculation of the OR of the HR and forest plots was calculated using the Metan package of the statistical program Stata®, version 14.

## Results

### Selection in the literature

**Fig. 1:**
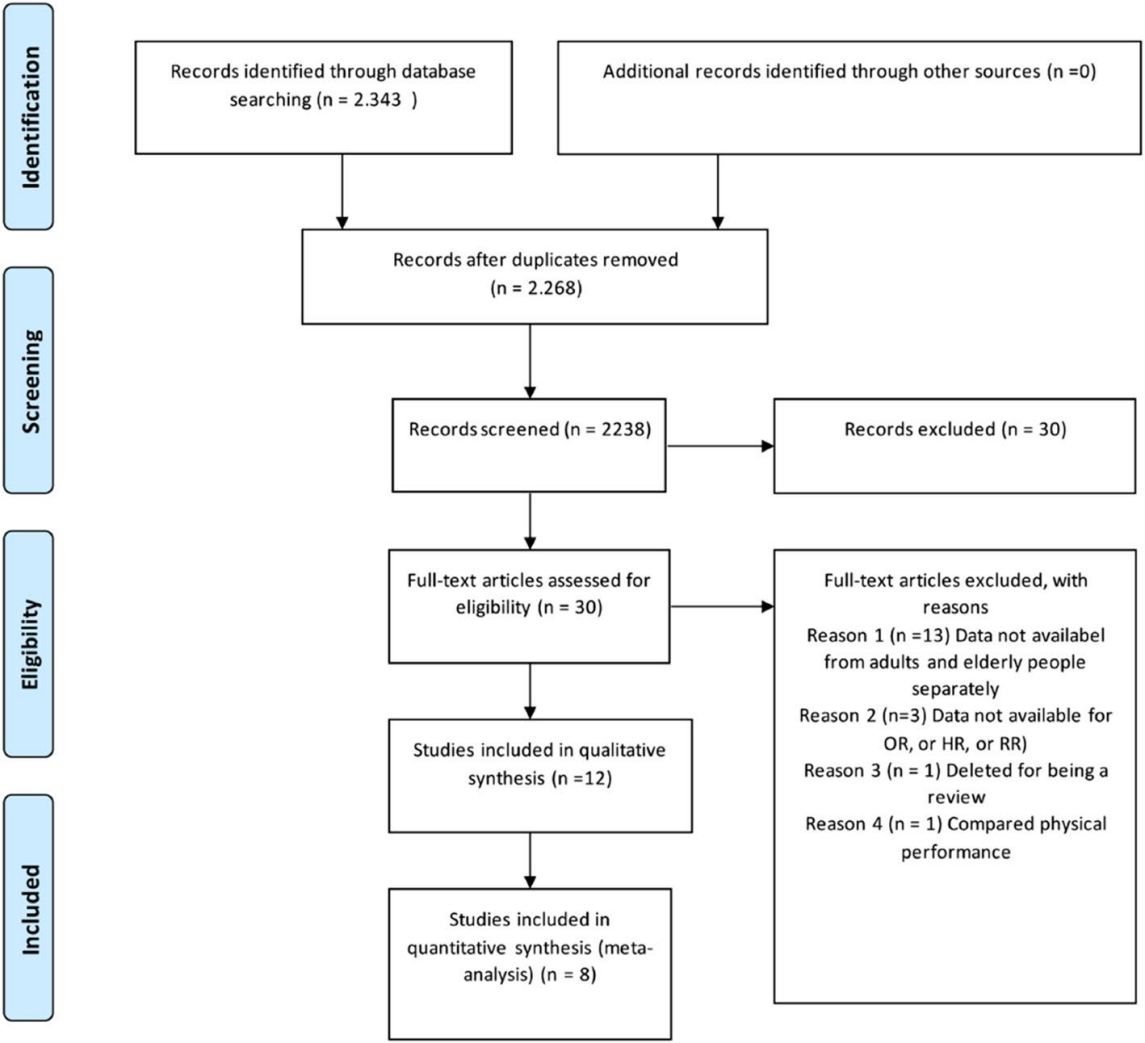
Flow diagram of preferred reporting items for systematic review and meta-analyses of the study selection process (PRISMA). Modified from: Moher et al. The PRISMA Group (2009). Preferred Reporting Items for Systematic Reviews and Meta-Analyses. The PRISMA Statement. PLoS Med 6(7): e1000097. doi:10.1371/journal.pmed1000097

The electronic search identified 2,343 potentially relevant studies. After reading the title and abstract, we excluded 2,238 publications for the following reasons: duplicate articles, study design, the sample did not include DM2 or older adults, or did not assess falls. After reading the abstract, 30 studies remained. Of this total, 18 were excluded for not presenting data on the ages of the younger and older adults separately; three articles were excluded for not presenting outcome measures, such as OR, HR, and RR; one article was excluded for being a review; and one article was excluded for comparing only physical performance. No attempt was made to access unpublished studies or other ‘grey’ literature. After we applied the inclusion and exclusion criteria, 12 publications were included, of which 8 studies were included in the meta-analysis (See Fig. 1 below).

### Study characteristics

The 12 included studies were published from 2002 to 2019 and conducted in eight different countries. The designs adopted in the studies were as follows: cohort [20, 25–27]; prospective cohort [28–31]; cross-sectional [19, 32]; retrospective cross-sectional [33]; prospective cross-sectional [34].

The sample sizes ranged from 77 to 22,200 individuals, and a total of 40,778 participants were identified and analyzed in the present review. The duration of follow-up was from 3 months to 10 years. The ages of the participants ranged from 60 to 101 years. The samples in ten studies included both older men and women, while two studies included only older women in their sample [28, 29]. The general characteristics of the included studies are listed in Table 1 (See file Table 1). Additional information about the characteristics of the cohort and cross-sectional studies are presented in Additional files for Review (Additional file 2: Table S2 and Additional file 3: Table S3 respectively).

**Table 1:** Baseline characteristics of the included studies

**Study/year**	**Region**	**Design**	**Population**	**Subject (%women)**	**Age/ Range of mean**	**Ascertainment of falls**	**Falls number (%)**	**Follow-up duration**	**Punctuation** **NOS**	**DM diagnosis (DM number)**
Schwartz et al. (2002) [32]	USA	Prospective cohort study	Community	9.249 (100% women)	73.5 ± 5.0	Postcard / phone	1,640 (18%)	2 years	7	Self-reports (629)
Maurer et al (2005) [30]	USA	Prospective cohort study	Long-stay institution	139 (84% women)	88 ± 7	Berg balance scale	49	Range 97-8854 days	8	Prescription of oral hypoglycemic agent or insulin therapy (extracted from medical records) (18)
Volpato et al (2005) [29]	USA	Cohort	Community	1.002 (100% women)	75.3 ± 6.5	Questionnaire	26,5	3 years	8	Specific disease investigation algorithm developed for this study (136)
Tilling et al. (2006) [35]	UK	Cross-sectional prospective	Hospital	77 (58.5% women)	73	Questionnaire	39%	5 months	5	Self-reports (77)
Schwartz et al. (2008) [28]	USA	Cohort	Community	3.075 (44.6% women)	73.6 ± 2.7	Questionnaire /self-reports	23%	5 years	8	Self-report, use of hypoglycemic medication or an elevated fasting glucose level (≥126 mg/dl) or 2-hour oral glucose tolerance test (≥200 mg/dl) (719)
Pijpers et al. (2011) [20]	NL	Cohort	Community	1.145 (49.8% women)	75.4 ± 6.5	Calendar	232 (20,3%)	3 years	8	Self-report and use of glucose-lowering medication (85)
Roman de Mettilinge et al (2013) [31]	BE	Prospective cohort	Community	199 (68.3% women)	76.9 ± 9.4	Questionnaire	56 (28,4%)	12 months	6	General practitioner or specialist physician confirmed the presence or absence of DM (104)
Yau et al (2013) [33]	USA	Prospective cohort	Community	3.075 (52% women)	73.7 ± 2.8	Medical record	293	10 years	7	Self-reported medical diagnosis, self-reported use of antidiabetic medications, elevated fasting glucose level (≥ 126 mg/dL) or elevated levels on a 2-hour oral glucose tolerance test (≥ 200 mg/dL) (719)
Bruce et al. (2015) [19]	AU	Cross-sectional	Community	186 (50% women)	70.3 ± 10.1	Questionnaire	39 (20,9%)	-*	4	Self-report and fasting glucose levels (186)
Chiba et al. (2015) [36]	JP	Cross-sectional retrospective	Community	211 (70,88% women)	76.2 ± 6.8	Questionnaire	62	Every 3 months for 3 years	5	Self-reports (168)
Randolph et al (2019) [27]	USA	Cohort	Community	22.200 (63.3% women)	78.3 ± 6.9	Medical record	411	5 years	6	Medical record (11.000)
Rashedi et al (2019) [34]	IR	Cross-sectional	Community	220 (58% women)	69.82 ±9.9	Questionnaire	77 (38,5%)	-*	3	Medical record (220)

Source: Research data, 2022. -*, Did not provide data on the duration of follow-up

*DM* Diabetes Mellitus, *NOS* New Castle Ottawa

As can be seen in Table 1, a population of older adults was studied in 10 of the included studies. Two other studies included older adults from specific settings: the hospital setting and the long-term care facility setting. Furthermore, the number of subjects varied widely (from 77 to 22,200), as did the methods of defining a diagnosis of DM, ranging from self-reports only [27, 30, 33]; combined self-reports and laboratory tests [19, 20, 26, 28, 31]; medical records [25, 32]; confirmation by a physician [29]; or using an algorithm [27].

The classic definition of the outcome fall, which is well established in the literature [14] was adopted in 3 studies [20, 29, 33], while Schwartz et al. [30] added “falling and hitting an object such as a table or a ladder” to this definition.

### Methodological quality of the included studies

Eleven studies presented a low risk of bias and only the study by Rashedi et al. [32] presented a high risk of bias. The complete evaluation of the studies according to the Newcastle-Ottawa Scale (NOS) is described in Table 2* - Quality Assessment and Risk of Bias of included studies. *(See file Table 2)*.*

**Table 2:** Quality assessment and risk of bias of included studies

Cross-sectional studies											
Author	Year		Selection		Comparability		Outcome			Score	Quality
		Representativeness	Selection	Ascertainment	Adjustment for confounders		Assessment		Response rate		
Bruce	2015	*	*	*	*		-		-	4	High
Chiba	2015	*	*	*	*		*		-	5	High
Rashedi	2019	*	-	*	*		-		-	3	Low
Tilling	2005	*	*	-	*		*		*	5	High
Cohort studies											
Author	Year		Selection		Comparability		Outcome			Score	Quality
		Representativeness	Selection	Ascertainment	Outcome	Adjustment for confounders	Assessment	Duration	Completeness of follow-up		
Maurer	2005	*	*	*	*	*	*	*	*	8	High
Pijpers	2011	*	*	*	*	*	*	*	*	8	High
Randolph	2019	*	*	-	-	*	*	*	*	6	High
Roman de Mettilinge	2013	*	*	*	*	*	-	*	-	6	High
Schwartz	2002	*	*	*	*	*	*	*	-	7	High
Schwartz	2008	*	*	*	*	*	*	*	*	8	High
Volpato	2005	*	*	*	*	*	*	*	*	8	High
Yau	2013	*	*	*	*	*	*	*	-	7	High

Source: Research data, 2022.

### Falls outcome assessment method

The preferred method used to assess the “falls” outcome in the studies was a questionnaire, which was used in 7 studies [19, 26, 27, 29, 32–34]. One study used the Berg Balance Scale [28], another study used a postcard [30], two studies used medical records [25, 31], and finally one used a calendar [20].

### Risk factors associated with falls

Twelve studies, including 14,061 older adults with T2DM, reported 1,394 falls. The HR and OR values for the risk factors for falls and the adjustments for covariates of the cohort and cross-sectional studies are shown in Tables S2 and S3 (Additional file 2: Table_S2 and Additional file 3: Table S3 respectively).

For the cohort studies [20, 25–31], participants with T2DM had a higher incidence rate of falling, recurrent falls, TCA/GABA-analog use, insulin use, and being female. The risk factors for falls in these studies were insulin use, followed by T2DM and medication use (Table S2).

The risk factors for falls in older adults in cross-sectional studies varied widely among the different studies: fear of falling, age, medication use, hypoglycemia, gait problems, body balance difficulty, hypotension, and older women (Table S3).

Multiple falls were reported in two studies [29, 33], with Chiba et al. showing that hypoglycemia and the Fall Risk Index were significant for multiple falls in diabetic patients. Recurrent falls were more prevalent in individuals with T2DM [20] and women on insulin therapy [27, 30].

The investigation of peripheral neuropathy as a risk factor for falls was reported in a single study, where recurrent falls and loss of pressure sensitivity were independently associated with the risk of falling more than once a year and accounted for 3-6% of the relation between diabetes and falling [30]. Visual impairment is another complication of DM that is frequently reported as a risk factor for older adults in the general population. However, we did not find studies investigating visual impairment and/or the presence of retinopathy as risk factors for falls in older adults with T2DM.

High glycated hemoglobin (HbA1C >7%) was a risk factor for falls, as well as dependence on a walking aid, such as a cane [34].

### Association between diabetes mellitus and risk of falls: results of meta-analysis

**Fig. 2:**
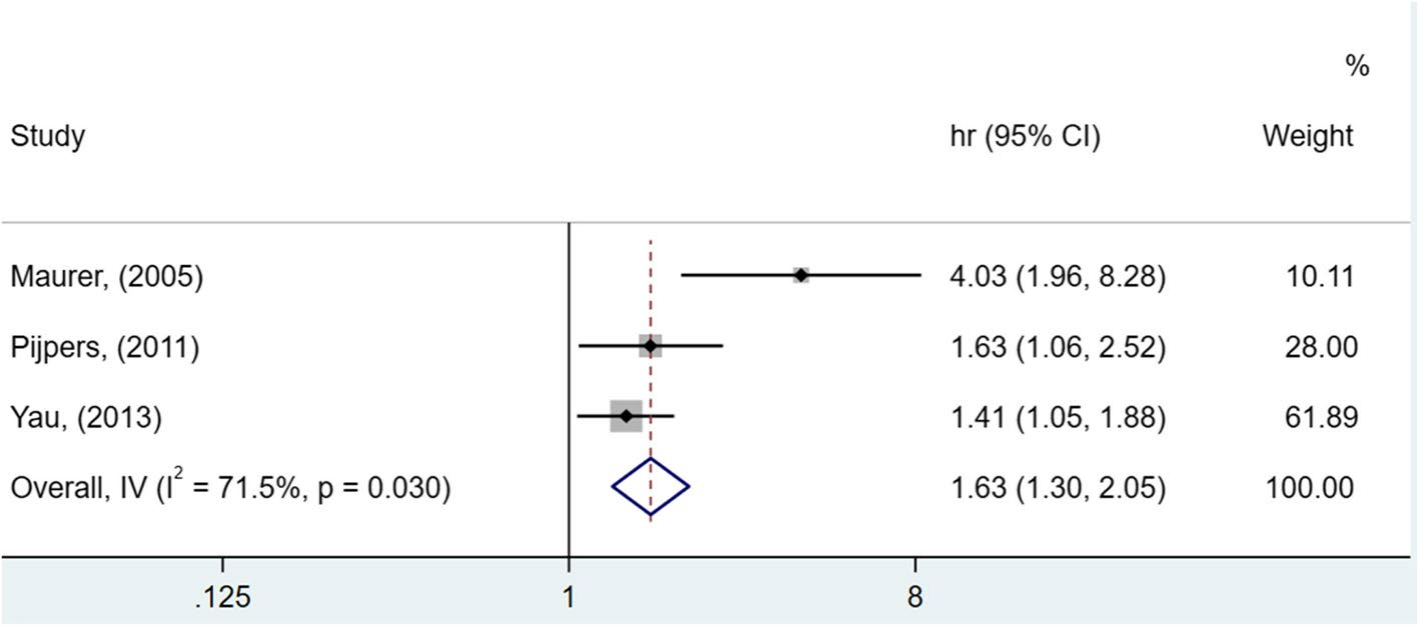
Forest plot showing increased risk of falls among elderly people with diabetes compared to non-diabetics (HR, 95% confidence interval)

**Fig. 3:**
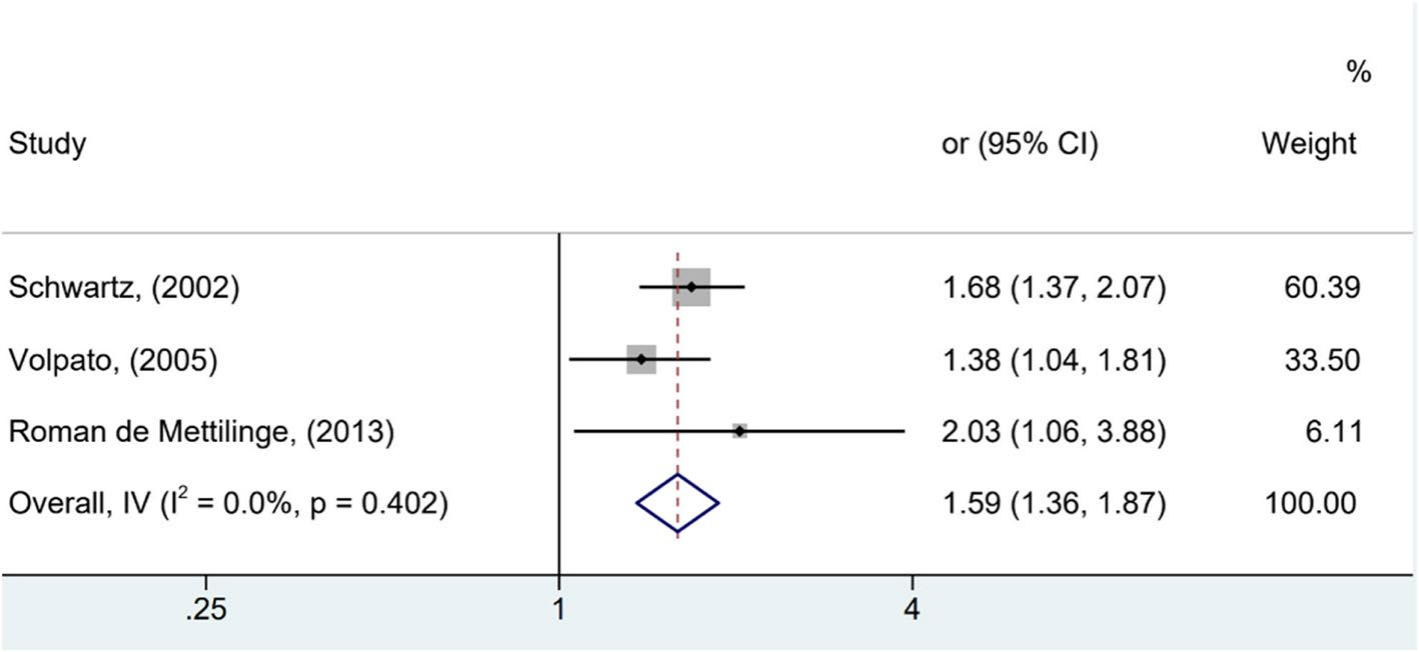
Forest plot showing increased risk of falls among elderly people with diabetes compared to non-diabetics (OR and 95% CI, random model)

**Fig. 4:**
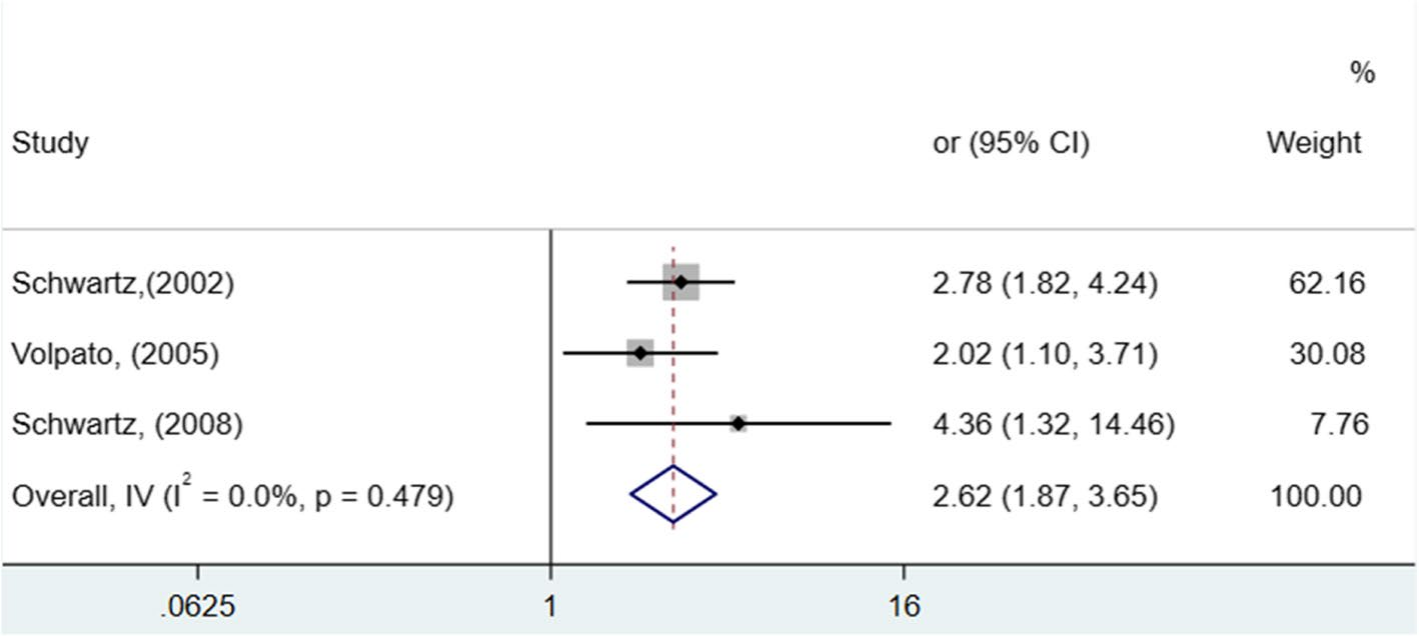
Forest plot showing increased risk of falls among elderly people with insulin-dependent diabetes compared to non-insulin-dependent diabetics (HR and 95% CI, random model)

As observed in Figs. 3 and 4, significant differences were identified among the analyzed studies. See Figs. 2, 3 and 4.

In Fig. 2, the risk of older adults with T2DM developing outcome falls is 63% higher than older adults without diabetes (HR 1.63; 95% CI [1.30 - 2.05]). The heterogeneity of this analysis was considerable (I^2^ = 71.5%). The chance of falls in older adults with T2DM is 49% higher compared to those without T2DM (OR 1.49; 95% CI [1.29 - 1.72]), with moderate heterogeneity (I^2^ = 36.4%), as shown in Fig. 3).

The chance of falls in older adults with T2DM who take insulin is 162% higher (OR 2.62; 95% CI [1.87 - 3.65]), with heterogeneity, as shown in Fig. 4) (I^2^ = 0.0%; low). No results related to diabetic polyneuropathy were found in the studies.

## Discussion

This meta-analysis revealed that older adults with T2DM have a higher risk of falls compared to non-diabetics [20, 26–29, 31]. Another important finding was the higher propensity of falls in insulin-dependent older adults than in non-insulin-dependent older adults [26, 27, 30]. It is known that older adults with T2DM present several complications due to the complexity of the disease, an increased risk of comorbidities, advancing functional decline, and the concomitant risk of frailty and falls [36].

The use of insulin is especially concerning in older adults because of the potential for hypoglycemic events and the increased risk of falls [35]. In the study of Volpato [27], the percentage of recurrent falls was particularly high for women with T2DM on insulin therapy (59%) and in the study of Schwartz [30], a higher proportion of women with T2DM fell more than once a year or more than twice a year compared to non-diabetic women. The highest risk of falls was observed in older women with T2DM who used insulin [35].

These findings are confirmed in the study of Yau [31], since participants who used insulin had a significantly higher risk of hospitalization due to a damaging fall than those who were non-diabetic.

In the meta-analysis, we did not observe an association between T2DM and medication use. Several studies included in the systematic review indicated that older adults with DM and who use medication [17, 24, 29, 30] present an increased risk of falls. The authors of longitudinal studies reported intrinsic risk factors for falls: age, abnormal gait pattern and body balance, and diabetes mellitus. Medication and insulin use were regarded as extrinsic factors for falls.

Other authors have also reported the use of medication and insulin as extrinsic factors associated with falls in older adults with T2DM [17, 24, 29, 37]. This association can be justified by the side effects of polypharmacy and drug interactions [38].

Accordingly, polypharmacy is frequently mentioned in the older adult population and is therefore not restricted to older adults with T2DM [39]. These effects of medication use were associated with recurrent falls and an increased risk in the domains of body balance and mobility, psychological risk factors, and sensory and neuromuscular function, and were recognized as risk factors for falls [37].

The aging process and some chronic diseases, which are related to oxidative stress, low-grade inflammation, and insulin resistance represent conditions that increase with age, leading to frailty. Diabetes and frailty are two conditions that are often found in older patients [40].

The endothelial dysfunction could explain the relationship between DM and frailty. The transition from pre-frailty to frailty triggered by hyperglycemia in hypertensive older adults could depend on the increased endothelial dysfunction [41]. Furthermore, frail elderly with hyperglycemia presented more physical impairment than normoglycemic frail elderly patients, and glycemia was strongly associated with gait speed [42].

Another finding referred to in the studies in this systematic review was the association between the presence of hypoglycemia and an increased risk of falling, worsened by the presence of neuropathy, impaired vision, and decreased cognitive and physical performance [43]. This finding was confirmed in older adults with T2DM in which hypoglycemia was significantly associated with falls (two or more per year) [33].

In this context, low glycemia increases the risk of frailty and functional decline in older people with type 2 diabetes [44].

It is interesting to observe that, both hypoglycemia and hyperglycemia during hospital stays are correlated with an increased risk for falls in the hospitalized population.

The presence of diabetes, use of insulin, or glucose variability were mentioned as potential risk factors for falls inside the hospital [45].

Different metabolic phenotypes of frailty could explain this apparent contradiction. Dysglycaemia (high and low glycemia) increases the risk of frailty in older people with diabetes. Frailty is heterogeneous and has a metabolic spectrum that begins with an anorexic malnourished frail phenotype and extends to the sarcopenic obese phenotype [46].

Falls in older adults are associated with morbidity and mortality as they can lead to serious injuries [39]. The higher prevalence of complications involving visual impairment and peripheral neuropathy was associated with an excessive risk of falls due to poor glycemic control [37]. The deficit in body balance and increased risk for falls in older adults with type 2 DM is often attributed to peripheral neuropathy [40]. In the current review, this hypothesis could not be investigated, as only the study by Schwartz et al. (2002) [30] highlighted that peripheral neuropathy was reported in association with falling more than once a year and was linked to decreased sensitivity to vibration and loss of sensitivity to pressure. This loss of pressure sensitivity was associated with the risk of falls occurring more than once a year.

In fact, DM-related complications feature loss of proprioceptive and tactile information and increased static standing postural sway, due to diabetic polyneuropathy (DPN). Diabetics with neuropathy present body balance impairment and this is associated with sensory deficits, indicating poor postural control. In DPN there is an additional decrease in reaction time with a delayed response to postural change and loss of muscle strength secondary to increased muscle atrophy [43].

Fear of falling was another factor associated with the risk of falls mentioned by the authors included in this review, because of which diabetic older adults restrict their participation in indoor and outdoor activities [19]. Thus, intrinsic, extrinsic, and behavioral factors contribute to the risk of falls in older adults. Initial and recurrent falls play an important role in intrinsic factors, as they often reduce physical activity and mobility in older adults [47]. The very visual and proprioceptive impairment found in advanced cases of DM reduces safety while walking. By restricting their participation in activities, reducing their living space, and reducing their interactions with challenging environments, older adults may end up entering a vicious cycle, where falls and disabilities lead to functional limitations and further falls [48].

Another consequence of poor quality of regulation of glycemic levels is the reduction of attention and poor physical performance reported in a case-control study enrolling diabetic patients (Type 1 DM and T2DM) under normal and hyperglycemic conditions. During transient hyperglycemia, the mean response time to nonverbal stimuli, and the traveled distance of the center of pressure during tandem gait test were significantly worse than normoglycemic conditions [49].

Maurer and Rashedi [28, 32] reported that altered gait patterns are a risk factor for falls in diabetic older adults. This change in gait pattern affects peripheral sensory and motor function, which is related to an increased risk of falling [43]. Diabetic retinopathy leads to a loss of contrast sensitivity and depth perception, which could lead to an increased risk of falls. However, in the current review, this finding was not reported in any of the included studies.

Another risk factor for falls in the elderly not mentioned in the studies included in this review was sarcopenia and the resulting muscle weakness. It is important to highlight that T2DM patients presented lower muscle performance and strength compared with euglycemic subjects and have an increased risk of sarcopenia compared with euglycemic subjects [50]. The presence of low muscle strength is a key characteristic of sarcopenia and poor physical performance is indicative of severe sarcopenia [51].

Falls were reported by questionnaires, a calendar, medical records, self-reports, or by the Berg Balance Scale. There is no consensus in the literature about the best tool for fall detection [52].

In the present review, falls in community-dwelling older adults were reported in 10 studies [19, 20, 25–27, 29–33] and these may have been caused by the risk factors that most affect this scenario, such as environmental factors, pharmacological agents, cognitive factors, and physiological factors [53]. The falls occurred in the hospital setting in only two studies [28, 34]. Hospital falls are a frequent and worrisome problem in the world and prevention strategies that should be more widely implemented are the education of physicians, modification of the environment, assistive devices, hospital systems, and medication reviews [54]. As we have noted, in both hospital and community settings, intrinsic and extrinsic factors may contribute to an increased risk of falls in the population of older adults with T2DM.

In the current review, most of the studies were prospective [28–31] cohort studies that showed follow-up of falls. Recent studies have used a 12-month [55–57] follow-up period, while in the current review, the follow-up period ranged from 3 months to 10 years.

The main limitation of this systematic review was the lack of studies that presented data regarding falls in adults and older adults separately. We found great heterogeneity in the methods adopted among the selected articles, such as differences in the duration of follow-up, number of participants, exclusion criteria, study designs, different population settings, different objectives among the studies, different definitions of falls, and different diagnostic criteria for the diagnosis of DM.

Other factors that could explain the increased risk of falls in older adults with DM should be investigated by further studies, for example, elevated glycated hemoglobin, high blood pressure, and the presence or absence of hypoglycemia, diabetic polyneuropathy, or diabetic retinopathy.

Considering these consequences of T2DM, the adoption of preventive strategies is mandatory, especially if we consider a significant age-dependent decline in the associations of obesity with hyperglycemia and dyslipidemia in men and women [58].

Interestingly, the main contribution of this study was to show that the same strategies used for treatment are the main risk factors for falls in diabetic older patients. This reinforces the importance of education in the rational use of medications and insulin, together with nutritional support and physical activities that may contribute to a decrease in the risk of falls in older adults with T2DM. Another result of these preventive measures would be a reduction in healthcare costs, considering the high level of care required and the increased length of hospital stays associated with falls in this population [54, 59].

## Conclusion

Older adults with T2DM have a higher risk of falls compared to non-diabetics. Among older adults with T2DM, some factors associated with a higher risk of falls were the use of insulin or other medications. General frailty also played a role.

Healthcare providers should be educated in the judicious use of medications and insulin to avoid iatrogenic falls. Physical therapy, adequate nutrition, and other general measures demonstrate the potential to decrease the burden on the healthcare system of complications arising from falls in older adults with T2DM.

### Supplementary Information

**Additional file 1: Table S1.** Search strategy on databases.

**Additional file 2: Table S2.** Characteristics of included cohort studies.

**Additional file 3: Table S3.** Characteristics of included cross-sectional studies.

**Additional file 4: Table S4.** Detailed information about criteria considered in each item of the “New Castle Ottawa Scale”(NOS) for appraisal of the risk of bias of cohort and cross-sectional studies.

## Data Availability

The datasets used and/or analyzed during the current study are available from the corresponding author upon reasonable request. We agree to share the output of the analysis from meta-analysis and tables containing the data extracted from studies. Detailed information about criteria considered in each item of the “Newcastle Ottawa Scale” for appraisal of risk of bias of cohort and cross-sectional studies is available in the file “Additional file 4: Table S4” in the Additional Files for Review section.

## Abbreviations

DM: Diabetes Mellitus
DPN: Diabetic polyneuropathy
GABA: Gamma-aminobutyric acid
HbA1C: High glycated hemoglobin
HR: Hazard Ratio
Newcastle: Ottawa (NOS)
OR: Odds Ratio
PRISMA: Criteria of the Preferred Reporting Items for Systematic Reviews and Meta-Analyses
RR: Relative risk
T2DM: Type 2 Diabetes Mellitus
TCAs: Tricyclic antidepressants
