# Supplementary material for: Risk factors for falls in older adults with diabetes mellitus: systematic review and meta-analysis
Source: BMC Geriatr. 2024 Feb 28;24:201. doi: 10.1186/s12877-024-04668-0 (PMC10900672; doi:10.1186/s12877-024-04668-0)
Supplement: Supplementary file 1 — Additional file 1: Table S1. Search strategy on databases. [file 12877_2024_4668_MOESM1_ESM.docx]

**Supplemental File 1** **- Search Strategies**

| **Data base** | Search Strategies |
| --- | --- |
| PubMed | #1 "aged"[MeSH Terms] OR "aged"[All Fields]  # 2 "diabetes mellitus, type 2"[MeSH Terms] OR "type 2 diabetes mellitus"[All Fields] OR "diabetes mellitus type 2"[All Fields]  # 3 "accidental falls"[MeSH Terms] OR ("accidental"[All Fields] AND "falls"[All Fields]) OR "accidental falls"[All Fields]  # 4 ("aged"[MeSH Terms] OR "aged"[All Fields]) AND ("diabetes mellitus, type 2"[MeSH Terms] OR "type 2 diabetes mellitus"[All Fields] OR "diabetes mellitus type 2"[All Fields])  # 5 ("accidental falls"[MeSH Terms] OR ("accidental"[All Fields] AND "falls"[All Fields]) OR "accidental falls"[All Fields]) AND (("aged"[MeSH Terms] OR "aged"[All Fields]) AND ("diabetes mellitus, type 2"[MeSH Terms] OR "type 2 diabetes mellitus"[All Fields] OR "diabetes mellitus type 2"[All Fields])) |

Source: Research data, 2022.

| Cinahl | S1 (MM "Diabetes Mellitus, Type 2")  S2 (MH "Aged+")  S3 (MH "Accidental Falls")  S4 ((MH "Accidental Falls")) AND (S1 AND S2)  S5 (((MH "Accidental Falls")) AND (S1 AND S2)) AND (S3 AND S4) |
| --- | --- |

Source: Research data, 2022.

| Web of Science | #1 diabetes mellitus type 2  #2 aged  #3 accidental falls  #4 #1 AND #2  #5 #3 AND #4 |
| --- | --- |

Source: Research data, 2022.

| Scopus | 1 ( diabetes AND mellitus AND type 2 )  2 ( aged )  3 ( accidental AND falls )  4 ( diabetes AND mellitus AND type 2 AND aged )  5 ( diabetes AND mellitus AND type 2 AND aged AND accidental AND   falls ) |
| --- | --- |

Source: Research data, 2022.

| Cochrane Library | #1 Diabetes Mellitus Type 2  #2 Aged  #3 Accidental falls  #4 #1 AND #2  #5 #3 AND #4 |
| --- | --- |

Source: Research data, 2022.
