# Supplementary material for: Risk factors for falls in older adults with diabetes mellitus: systematic review and meta-analysis
Source: BMC Geriatr. 2024 Feb 28;24:201. doi: 10.1186/s12877-024-04668-0 (PMC10900672; doi:10.1186/s12877-024-04668-0)
Supplement: Supplementary file 2 — Additional file 2: Table S2. Characteristics of included cohort studies. [file 12877_2024_4668_MOESM2_ESM.docx]

**Table**

Main results of the Cohort studies.

| **Study/year** | **Risk factors for falls** | **HR/OR (95% CI) p valor** | **Adjustment for covariates** |
| --- | --- | --- | --- |
| Schwartz et al. (2002) ^(32)^ | Insulin, age | [OR] 1,68 [IC 95% 1,37–2,07] | Age, medical history, medications, visual acuity, cognitive function, depression, walking speed |
| Maurer et al (2005) ^(30)^ | Diabetes mellitus, poor gait and balance | Diabetes mellitus ([IC] 95%, HR= 4,03 (1,96-8,28); gait and balance (IC 95%, HR = 5,26 (1,26-22,02) | Diabetes mellitus, gait and balance |
| Volpato et al (2005) ^(29)^ | Insulin therapy, overweight, lower extremity pain, and an unsatisfactory lower extremity summary performance score | Insulin (OR ajustado 2,73; IC de 95%, 1,61–4,63) | Obesity, adjudicated knee osteoarthritis, and stroke |
| Schwartz et al. (2008)^(28)^ | Insulin, loss of discrimination to light touch, and low amplitude peroneal nerve response | OR 4,36 [IC 95% 1,32-14,46]) | Age, diabetes complication, vision, blood pressure, insulin, peripheral nerve function, physical performance, laboratory measurements |
| Pijpers et al. (2011) ^(20)^ | Diabetes mellitus, more medications, higher levels of pain, poorer self-perceived health, less physical activity and grip strength, more limitations in ADLs, lower physical performance in limbs and cognitive impairment | Risk of recurrent falls with DM [HR = 1,67 (IC de 95%: 1,11–2,51)] | Age, DM diagnosis, verification of recurrent falls, lifestyle, education level, comorbidity and medications, physical disability and general health, physical performance |
| Roman de Mettilinge et al (2013) ^(31)^ | Diabetes mellitus, urinary incontinence, walking with mobility devices, reporting falls in the previous year and reporting fear of falling | Diabetes (OR = 2,03, IC 95% 1,06–3,88); mediolateral  LOS displacement (OR = 0,70, IC 95% 0,49–0,99) | Age, medication, worse performance in handgrip strength, slower walking, with shorter strides and greater variability, lower mid-lateral limits of stability and worse performance on the MMSE |
| Yau et al (2013) ^(33)^ | Diabetes mellitus, insulin dependent | Diabetes [HR] 1,48 [IC 95% 1,12-1,95] | History of fainting, standing body balance score, cystatin C level for kidney function, and number of medications prescribed |
| Randolph et al (2019) ^(27)^ | Use of TCA/GABA-analog | Use of TCA/GABA-analog [HR] = 1,11; [IC] 95% = 1,03-1,20) | Alzheimer's disease and other dementias; arthritic conditions; inappropriate medication use |
