# Supplementary material for: Risk factors for falls in older adults with diabetes mellitus: systematic review and meta-analysis
Source: BMC Geriatr. 2024 Feb 28;24:201. doi: 10.1186/s12877-024-04668-0 (PMC10900672; doi:10.1186/s12877-024-04668-0)
Supplement: Supplementary file 3 — Additional file 3: Table S3. Characteristics of included cross-sectional studies. [file 12877_2024_4668_MOESM3_ESM.docx]

**Table**

Main results of cross-sectional studies.

| **Study/year** | **Risk factors for falls** | **HR/OR (95% CI) p valor** | **Adjustment for covariates** |
| --- | --- | --- | --- |
| Tilling et al. (2006) ^(36)^ | Poor diabetes control (HbA1c >7%), dependence on aid to work, previous stroke | Poor diabetes control (HR) = 7,83 (2,948–20,799), dependence on aid to work (HR) = 2.679 (1.998-3.593) e previous stroke (HR) = 1.929 (1.143-3.257). | -* |
| Bruce et al (2015) ^(19)^ | Advanced age, use of antidepressants and poor balance | History of recent falls is associated with age (OR: 1,65 (1,20-2,28); use of antidepressant (OR:2,14 (1,02–4,50);  Adjust for the above factors with regression ZINB: poor balance (IC 95%): 2,32 (1,15-4,70) | Age, antidepressant use, and limitation related to fear of outdoor activity |
| Chiba et al. (2015) ^(35)^ | Hypoglycemia | Hypoglycemia (OR 3,62, IC 95%: 1,242–10,534, P = 0,018).  Fall Risk (OR 1,2, IC 95%:1,010-1,425, P = 0,039) | Age, sex, presence of diabetes mellitus, GDS-15 and TUG test scores and presence of hypoglycemia |
| Rashedi et al (2019)^(34)^ | Gait problems, difficulty balance, hypotension, and medication above 3 | Gait problems (OR = 1,8, IC 95% =1,1 - 4,9), difficulty balance (OR = 2,1, IC 95% = 1,24 -7,12), hypotension (OR = 1,7, IC 95% =1,2 - 5,6), and medication above 3 (OR = 1,55, IC 95%=1,12 - 6,34) | Gait problems, difficulty balance, vision problems, neurological and cognitive impairments, osteoporosis, hypertension, hypotension, medications, and sleep quality |

Source: Research data, 2022. DM, Diabetes Mellitus; HR, hazard ratio; HbA1c, glycated hemoglobin; MMSE, Mini mental state exam; OR, odds ratio; TUG, test Timed Up and Go*;* -*, Did not show adjustment for covariates; ZINB, zero-inflated negative binomial regression; GDS-15, 15-item geriatric depression scale.
