# Supplementary material for: Risk factors for falls in older adults with diabetes mellitus: systematic review and meta-analysis
Source: BMC Geriatr. 2024 Feb 28;24:201. doi: 10.1186/s12877-024-04668-0 (PMC10900672; doi:10.1186/s12877-024-04668-0)
Supplement: Supplementary file 4 — Additional file 4: Table S4. Detailed information about criteria considered in each item of the “New Castle Ottawa Scale”(NOS) for appraisal of the risk of bias of cohort and cross-sectional studies. [file 12877_2024_4668_MOESM4_ESM.docx]

Additional_file_4_Table_S4. Detailed information about criteria considered in each item of the “New Castle Ottawa Scale” for appraisal of the risk of bias of cohort and crossectional studies.

| **Cohort studies** |
| --- |
| Note: A study can be given a maximum of one star for each numbered item within the Selection and Outcome categories. A maximum of two stars can be given for Comparability.  Selection  1. Representativeness of the exposed cohort with Diabetes Mellitus (DM)  a. Subjects representative of the average subjects aged 60 years and older with diabetes mellitus *  b. Not representative or no description  2. Selection of the non-exposed cohorts: subjects without Diabetes Mellitus drawn from the same population as the exposed cohort.  a. Yes *  b. No  c. No description of the derivation of the non-exposed cohort  3. Ascertainment of exposure: how diabetes mellitus diagnosis is made.  a. Diabetes mellitus diagnosed is made based on laboratory tests (fasting blood glucose, oral glucose tolerance test (TOTG), glycated hemoglobin (HbA1c), and self-report of a physician’s diagnosis * ^56,57^  b. Diabetes mellitus diagnosed is made based on laboratory tests (fasting blood glucose, oral glucose tolerance test (TOTG), glycated hemoglobin (HbA1c) self-report of a physician’s diagnosis and anti-diabetic medication use * ^56,57^  c. Diabetes mellitus diagnosed is made based on laboratory tests (fasting blood glucose, oral glucose tolerance test (TOTG), glycated hemoglobin (HbA1c), self-report of a physician’s diagnosis, anti-diabetic medication use and collecting data.  d. No description or unclear  4. Demonstration that fall outcome was not present at the start of study.  a. Yes*  b. No  Comparability  1. Comparability of cohorts on the basis of the design or analysis controlled for confounders  a. Study controls for age and/or sex*  b. Study controls for age and/or sex plus other factors*  c. Cohorts are not comparable on the basis of the design or analysis controlled for confounders.  Outcome  1. Assessment of fall outcome  a. Observed/assessed by a physician*  b. Medical/hospital records as the primary source*  c. Self-report  d. No description  2. Was follow-up long enough for fall outcomes to occur.  a. Yes, >= 12 months * ^2^  b. No, < 12 months  3. Adequacy of follow-up of cohorts  a. Complete follow up- all subjects accounted for *  b. Subjects lost to follow-up unlikely to introduce bias-number lost less than or equal to 20% or description of those lost suggested no different from those followed *  c. Follow up rate less than 80% and no description of those lost  d. Not described  *= one star |

Source: Research data, 2022.

| **Adapted for cross-sectional studies** |
| --- |
| Selection (Maximum 3 stars)  1. Representativeness of the sample  a. Subjects representative of the average subjects aged 60 years and older *  b. Not representative or no description  2. Selection of the non-exposed subjects: subjects without diabetes mellitus drawn from the same population as the exposed subjects  a. Yes*  b. No  c. No description of the derivation of the non-exposed subjects  3. Ascertainment of exposure: how diabetes mellitus diagnosis is made  a. Diabetes mellitus diagnosed is made based on laboratory tests (fasting blood glucose, oral glucose tolerance test (TOTG), glycated hemoglobin (HbA1c), and self-report of a physician’s diagnosis * ^56,57^  b. Diabetes mellitus diagnosed is made based on laboratory tests (fasting blood glucose, oral glucose tolerance test (TOTG), glycated hemoglobin (HbA1c) self-report of a physician’s diagnosis and anti-diabetic medication use * ^56,57^  c. Diabetes mellitus diagnosed is made based on laboratory tests (fasting blood glucose, oral glucose tolerance test (TOTG), glycated hemoglobin (HbA1c), self-report of a physician’s diagnosis, anti-diabetic medication use, and collecting data.  d. No description or unclear  Comparability (Maximum 2 stars)  1. The subjects in different outcome groups are comparable, based on the study design or analysis. Confounding factors are controlled.  a. Study controls for age and/or sex*  b. Study controls for age and/or sex plus other factors*  c. Study not controlling for any confounding factors.  Outcome (Maximum 2 stars)  1. Assessment of fall outcome  a. Observed/assessed by a physician*  b. Record linkage *  c. Self-report  d. No description  2. Response rate  a. Rate of sample loss <=20%*  b. Rate of sample loss >20%  c. Not stated  *= one star |
